# Supplementary material for: SMASH: Scalable Method for Analyzing Spatial Heterogeneity of genes in spatial transcriptomics data
Source: PLoS Genet. 2023 Oct 20;19(10):e1010983. doi: 10.1371/journal.pgen.1010983 (PMC10619839; doi:10.1371/journal.pgen.1010983)
Supplement: S1 Text — Section 1 discusses how to choose suitable kernel covariance matrices and combine the p-values corresponding to different kernel covariance matrices. Section 2 shows SPARK-X’s equivalence with the multiple linear regression model. Section 3 analyzes the null QQ plots of different methods in the real datasets. Section 4 discusses the severity of using non-positive definite (non-PD) kernel covariance matrices. We list and briefly describe the figures from S1 Text below. Fig A. Visualization of patterns of different kernel covariance matrices.Fig B. QQ-plots of different methods under null simulations in the real datasets.Fig C. QQ-plots with the observed and theoretical distributions of the SMASH test statistic with an unadjusted cosine kernel matrix.Fig D. QQ-plots with the observed and theoretical distributions of the SMASH test statistic with an adjusted cosine kernel matrix.Fig E. QQ-plots with the observed and theoretical distributions of the—log10(p)-values obtained using SMASH with all the kernel matrices. (PDF) [file pgen.1010983.s001.pdf]

Supplementary information for "SMASH: Scalable Method for Analyzing Spatial Heterogeneity of genes in spatial transcriptomics data"

Souvik Seal, Benjamin G. Bitler, and Debashis Ghosh[1], Souvik Seal[1], Benjamin G. Bitler[2], and Debashis Ghosh[3]

[1]Department of Public Health Sciences, Medical University of South Carolina, Charleston, South Carolina, USA

[2]Department of Obstetrics and Gynecology, School of Medicine, University of Colorado Denver Anschutz Medical Campus, Aurora, Colorado, USA

[3]Department of Biostatistics and Informatics, Colorado School of Public Health, University of Colorado Denver Anschutz Medical Campus, Aurora, Colorado, USA

1 Further details on the SMASH test statistic

1.1 Choice of kernel covariance matrices

In the main text, we define the SMASH test statistic for a gene k to have the following form,

$$T_k^{\text{SMASH}} \equiv \frac{tr(E_k K_{\mathcal{S}})}{N}; \quad E_k = y_k (y_k^T y_k)^{-1} y_k; \tag{1}$$

where $K_{\mathcal{S}}$ is any $N \times N$ kernel-based covariance matrix based on the locations S and y_k is the mean-standardized expression vector of the gene k. As mentioned in the main text, when additional covariates are present, we would replace y_k by $y_k^* = [I - P_X]y_k; P_X = X(X^T X)^{-1}X^T$, where X is the design matrix. We consider $K_{\mathcal{S}}$ to have following three forms:

1. The Gaussian kernel-based covariance matrix:

$$K_{\mathcal{S}} = \left[\left[\exp\left(-\frac{||s_i - s_j||^2}{2l^2}\right)\right]\right]_{N\times N} ; \quad ||s_i - s_j||= \sqrt{(s_{i1} - s_{j1})^2 + (s_{i2} - s_{j2})^2}$$

   for ten values of the lengthscale parameter l.

2. The cosine kernel-based covariance matrix of the form:

$$K_{\mathcal{S}} = \left[\left[\cos\left(\frac{2\pi||s_i - s_j||}{p}\right)\right]\right]_{N\times N}$$

   for ten values of the period parameter p.

3. The linear kernel-based covariance matrix of the form:

$$K_{\mathcal{S}} = g(S)(g(S)^T g(S))^{-1} g(S)^T$$

   where three choices of the coordinate-wise transformation g are considered:

   - An identity transformation i.e., $g(s_{i1}) = s_{i1}, g(s_{i2}) = s_{i2}$ for $i = 1, \ldots, N$, or, $g(S) = S$.

   - A Gaussian transformation as $g(s_{i1}) = \exp(-s_{i1}^2/2t_1^2), g(s_{i2}) = \exp(-s_{i12}^2/2t_2^2)$ for $i = 1, \ldots, N$, for five values of scale parameters t_1 and t_2.

- A cosine transformation as $g(s_{i1}) = \cos(2\pi s_{i11}/\phi_1), g(s_{i2}) = \cos(2\pi s_{i12}/\phi_2)$ for $i = 1, \ldots, N$, for five values of period parameters ϕ_1 and ϕ_2.

The Gaussian and cosine kernel-based covariance matrices have earlier been used in SpatialDE [6] and SPARK [5]. We follow SpatialDE to choose a set of fixed grid points for both the length-scale parameter l and the period p. In particular, we first obtain the minimum (d_{min}) and the maximum (d_{max}) values of the non-zero Euclidean distances across all pairs of spatial locations. Then, we extract ten equally-spaced values between $\log_{10}(d_{min}/2)$ and $\log_{10}(d_{max}/2)$. These values are then converted to the original scale by taking the power of ten and subsequently used as the values of l and p. Let us denote the test statistics corresponding to these twenty covariance matrices (10 each for the Gaussian and cosine kernels) as, $T_{kr}^{\text{SMASH}}, r = 1, \ldots, 20$. The linear kernel-based covariance matrices with transformed coordinates (g-transformation) have been earlier used in SPARK-X [8]. Following SPARK-X, we vary the transformation parameters, t_1, t_2, ϕ_1 and ϕ_2 to be the $20\%, 40\%, 60\%, 80\%$, and 100% quantiles of the absolute values of the x and y coordinates in the data. Let us denote the test statistic corresponding to these eleven (1 for the identity transformation and five each for the Gaussian and cosine transformations) linear kernel-based covariance matrices as, $T_{kr}^{\text{SMASH}}, r = 21, \ldots, 31$. We denote the p-values corresponding to each T_{kr}^{SMASH} as p_{kr}. Then, we can get SPARK-X's result by combining the p-values, $p_{kr}, r = 21, \ldots, 31$ using a Cauchy combination rule [3]. Note that the same Gaussian and cosine kernel-based covariance matrices ($r = 1, \ldots, 10$) are also used in SpatialDE [6] but not in SPARK-X. The linear kernel-based covariance matrix with identity transformation ($r = 21$) is used in both SpatialDE and SPARK-X, while the other transformations ($r = 22, \ldots, 31$) are uniquely considered in SPARK-X. We can get an approximate version of the SpatialDE (more in Section 1.2) p-value by combining $p_{kr}, r = 1, \ldots, 21$ using a simple minimum p-value approach as, $p_{k,comb1} = 21 * \min\{p_{k1}, \ldots, p_{k21}\}$. To combine the SPARK-X specific p-values ($r = 22, \ldots, 31$), we follow the same Cauchy combination rule [3] to get $p_{k,comb2}$. To construct the final p-value of SMASH, we perform a minimum p-value combination as, $p_{k,final} = 2 * \min\{p_{k,comb1}, p_{k,comb2}\}$. We do acknowledge that using two levels of minimum p-value combination can be conservative in some cases, which is partly observed in the null simulation

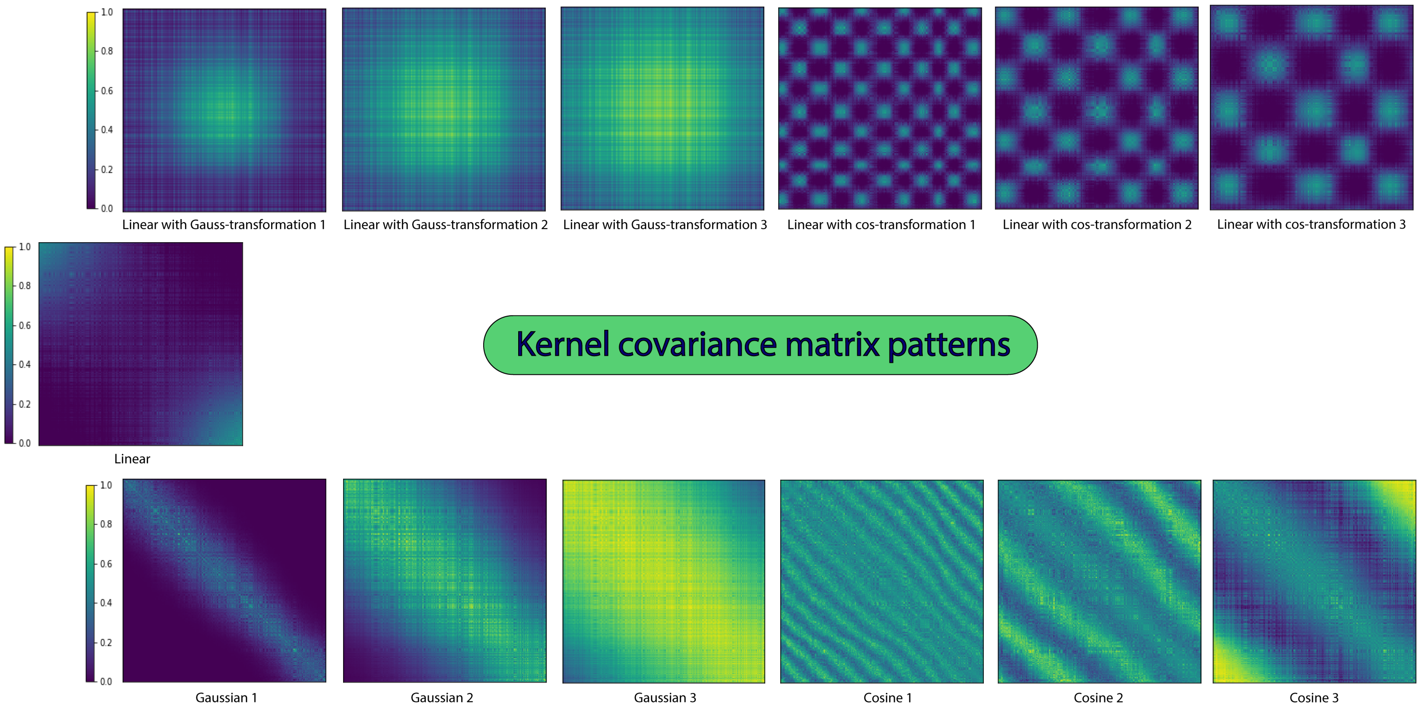

Fig A: The patterns of kernel covariance matrices for different choices of kernels, namely, linear, Gaussian, Cosine, linear on Gaussian-transformed coordinates, and linear on cosine-transformed coordinates. Three values of the associated hyper-parameter have been considered for every kernel (except linear), e.g., Gaussian 1, Gaussian 2, and Gaussian 3 correspond to three different choices of the length-scale parameter l, respectively. SMASH is capable of capturing all of these patterns in the gene expression, while SPARK-X captures only the patterns in the top row and the one in the middle.

studies performed using the real datasets in Section 3. However, our focus was on preventing the high degree of inflation in the p-values routinely observed in model-based methods like SpatialDE and SPARK. In Fig A of this text, we show the different patterns in the spatial domain every choice of kernel leads to.

1.2 Similarity with SpatialDE

From Equation (2) of the main text, recall that SpatialDE considers the following model,

$$y_k \sim N(\mu_k \mathbf{1}, \tau_k^2 \Sigma + \sigma_k^2 I)); \quad \Sigma = [[\Sigma_{ij}]]_{N \times N}; \quad \Sigma_{ij} = \exp\left[-\frac{||s_i - s_j||^2}{2l^2}\right];$$

SpatialDE optimizes two likelihood functions: one corresponding to the 'full' model shown above and the other corresponding to the reduced 'null' model without the $\tau_k^2\Sigma$ component in the covariance. Then, to test the null hypothesis, $H_0 : \tau_k^2 = 0$, it constructs a likelihood ratio test (LRT) comparing the optimized values of the above two likelihood functions. If y_k is mean-standardized i.e., $\mu_k = 0$, the corresponding score test statistic [1] can be shown to have the form,

$$\begin{aligned}
T_{score} &= y_k^T \Sigma y_k \\
&= tr(y_k y_k^T \Sigma) \\
&= (y_k^T y_k) tr(E_k \Sigma) \quad \text{since } (y_k^T y_k) \text{ is a scalar and } E_k = y_k (y_k^T y_k)^{-1} y_k, \\
&= N(y_k^T y_k) T_k^{\text{SMASH}} \quad \text{when } K_{\mathcal{S}} = \Sigma \text{ in Equation (1).}
\end{aligned}$$

2 SPARK-X's equivalence with a multiple linear regression model

Let us consider the following linear regression model with y_k as the dependent variable and the columns of the spatial location matrix S (x and y coordinates) as the predictors,

$$y_k = S\beta_k + \epsilon_k; \quad \epsilon_k \sim N(0, \sigma_k^2 I)$$

The fixed effect coefficients vector β_k is of length 2 (for 2D space) and its OLS estimate has the form, $\hat{\beta}_k = (S^T S)^{-1} S^T y_k$ with an estimated covariance matrix of $var(\hat{\beta}_k) = \hat{\sigma}_k^2 (S^T S)^{-1}$, $\hat{\sigma}_k^2 = y_k^T (I - D) y_k / N$; where $D = S(S^T S)^{-1} S^T$ from the main text. To test the null hypothesis, $H_0 : \beta_k = 0$, we

would consider the following Wald test statistic,

$$
\begin{aligned}
T &= \hat{\beta}_k^T (var(\hat{\beta}_k))^{-1} \hat{\beta}_k / N \\
&= y_k^T S(S^T S)^{-1}(S^T S)(S^T S)^{-1} S^T y_k/(N\hat{\sigma}_k^2) \\
&= y_k^T S(S^T S)^{-1} S^T y_k/(N\hat{\sigma}_k^2) \\
&= tr((y_k y_k^T)(S(S^T S)^{-1} S^T))/(N\hat{\sigma}_k^2) \\
&= (y_k^T y_k) tr(E_k(S(S^T S)^{-1} S^T))/(N\hat{\sigma}_k^2) \quad \text{since } (y_k^T y_k) \text{ is a scalar and } E_k = y_k(y_k^T y_k)^{-1} y_k^T, \\
&= [(y_k^T y_k)/\hat{\sigma}_k^2][tr(E_k D)/N] \quad \text{where } D = S(S^T S)^{-1} S^T \text{ from the main text,} \\
&= [(y_k^T y_k)/\hat{\sigma}_k^2] T_k^{\text{SPARKX}} \quad \text{where } T_k^{\text{SPARKX}} = tr(E_k D)/N \text{ from the main text.}
\end{aligned}
$$

Thus, we have shown that the SPARK-X test statistic T_k^{SPARKX} is proportional to the Wald test statistic obtained from a multiple linear regression model. However, the asymptotic distributional assumptions under null are different in both cases. Note that SPARK-X also replaces S by $g(S)$ where g is a coordinate-wise transformation as discussed in Section 1.1.

3 QQ-plots under null simulations

As mentioned in the main text, we performed null simulation studies to construct an empirical null distribution of the p-values for every method. With each of the four datasets, we randomly permuted the cell/spot coordinates five times and then applied the three methods, SMASH, SPARK-X, and SpaGene to obtain the respective p-values. The p-values were then transformed as $-\log_{10}(p\text{-value})$ and displayed against the expected values as quantile-quantile plots (QQ-plots) in Fig B of this text. We noticed that SMASH showed no sign of p-value inflation and was rather slightly conservative. It is expected since the minimum p-value combination rule we use, is known to be conservative [4] (see Section (1.1)). SpaGene produced slightly inflated p-values in the SCCOHT dataset while SPARK-X did not show any sign of inflation.

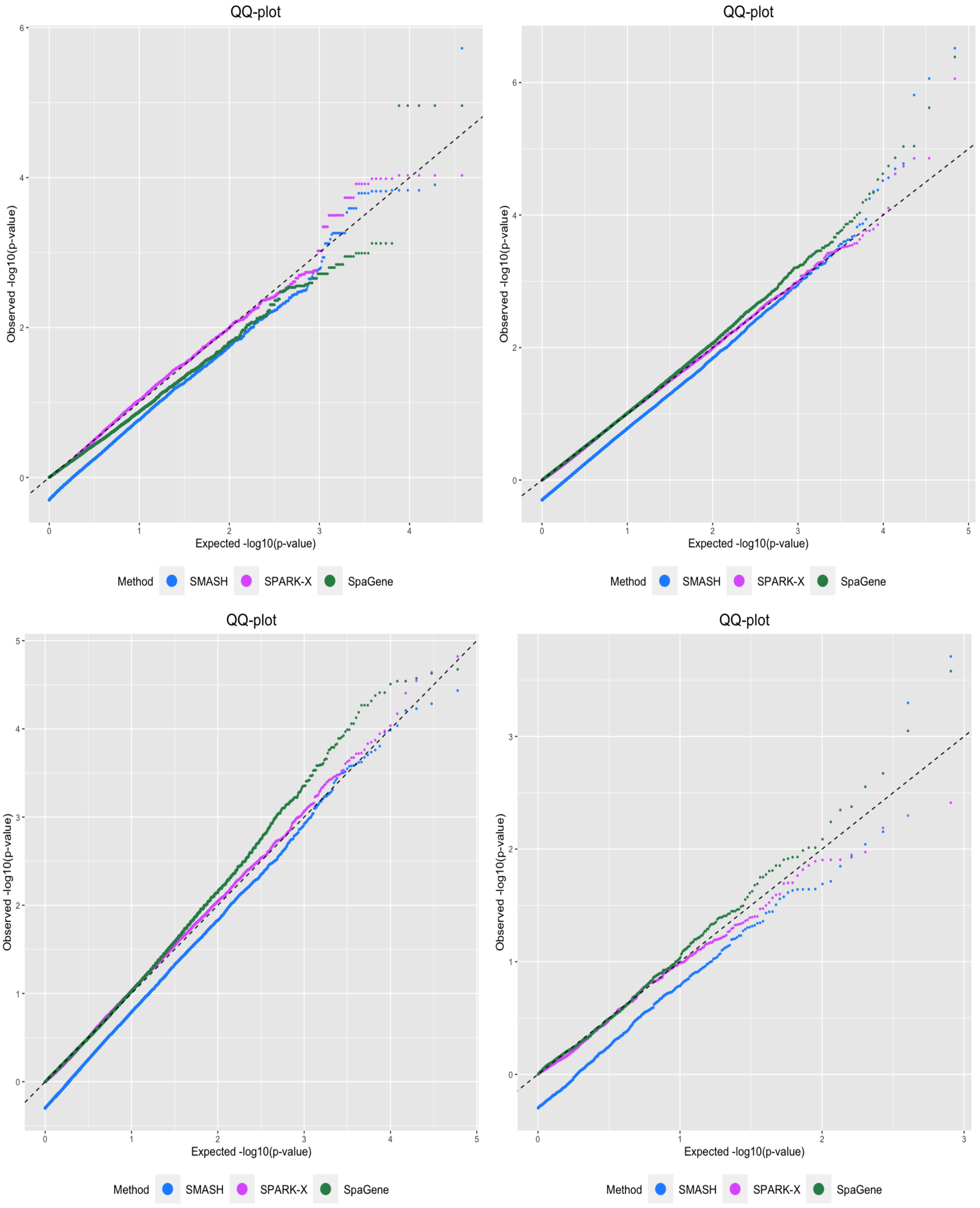

Fig B: Top left: Mouse cerebellum by Slide-seqV2, top right: Human DLPFC by 10X Visium, bottom left: SCCOHT by 10X Visium, bottom right: Mouse hypothalamus by MERFISH

4 Use of non-PD kernels

A problem with considering a cosine kernel covariance matrix is its lack of positive-definiteness. In our simulation model, we adjusted the cosine kernel covariance matrix by forcing the negative eigenvalues to be equal to 0, a strategy that is used in both SpatialDE and SPARK models [6, 5]. A crucial assumption behind our test is that the kernels $k_{\mathcal{Y}}$, $k_{\mathcal{S}}$ and consequently, the corresponding covariance or Gram matrices need to be positive-definite. Thus, the asymptotic distribution we have been considering, only holds if $K_{\mathcal{S}}$ is positive-definite. In Figs C and D of this text, we display the QQ plots of the empirical and theoretical distribution of our test statistic with unadjusted and adjusted cosine kernel covariance matrices, respectively. By adjustment we refer to replacing $K_{\mathcal{S}} = \sum_{i=1}^{N} \lambda_i U_i U_i^T$ by $K_{\mathcal{S}}^* = \sum_{i=1}^{N} \max\{\lambda_i, 0\} U_i U_i^T$, where λ_i and U_i denote the i-th eigenvalue and eigenvector, respectively. Quite expectedly, with an unadjusted cosine kernel matrix, the empirical distribution differed significantly from the theoretical distribution but the extreme values were below the $y = x$ line, indicating a conservative nature of the test. With an adjusted cosine kernel matrix, the distributions mostly agreed with each other. However, to adjust a cosine or periodic kernel matrix, we first need to compute its eigenvalues, a step requiring computational complexity of $O(N^3)$. Unlike traditional methods like SpatialDE and SPARK-X, we do not want to follow this route to avoid the computational burden. Instead, we argue that the simple unadjusted test statistic is conservative in nature and thus, can be usable in most practical scenarios. In Fig E of this text, we show the QQ plot of the observed and theoretical distributions of $-\log_{10}(p)$-values obtained by our aggregate test (i.e., p-values were combined for different kernel choices) in the same simulation setup. Notice that the test was conservative in all cases. In null simulations using all four real datasets as well, we noticed similar QQ plots (Fig B of this text). Another simplistic solution could be to drop the cosine kernels entirely from the analysis, following methods like SOMDE [2] and nnSVG [7]. Our *Python* package allows users to choose one of the three options: a) consider unadjusted cosine kernels, b) consider adjusted cosine kernels, and c) do not consider cosine kernels.

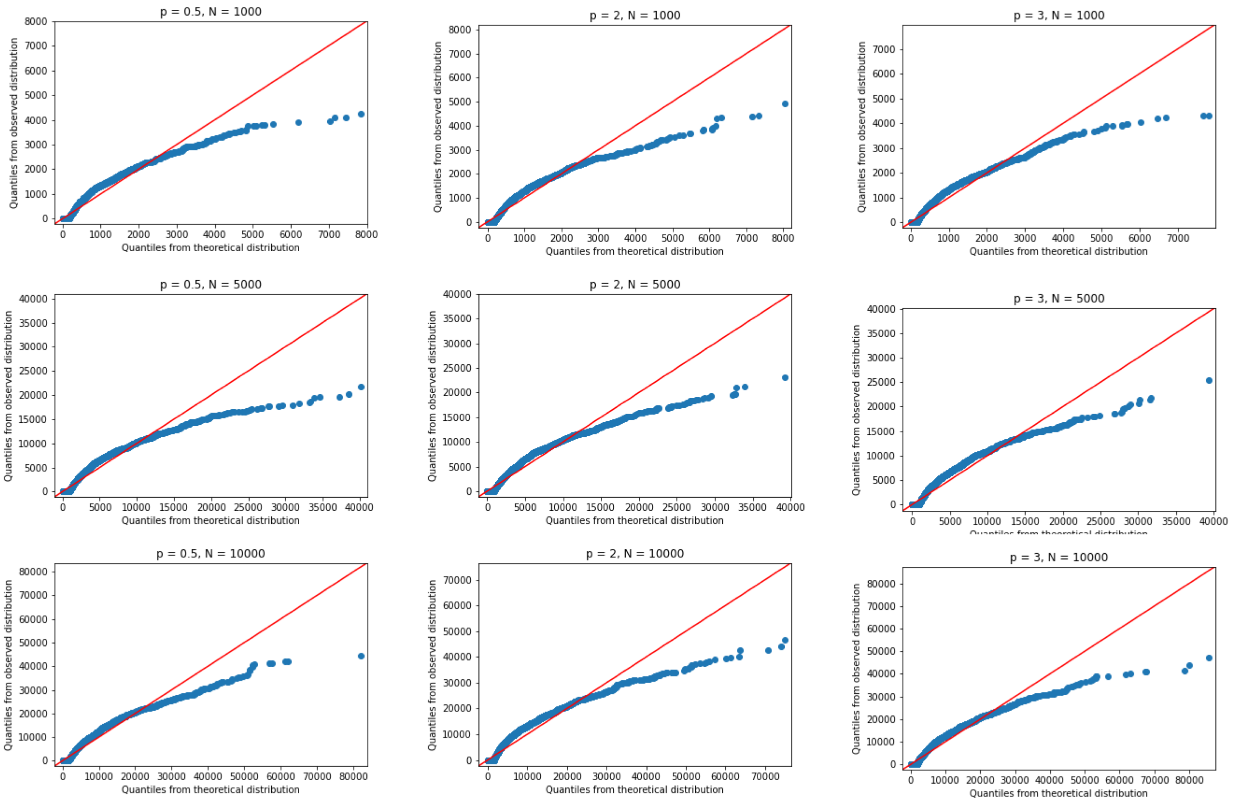

Fig C: The QQ plot of the observed and theoretical distribution of the test statistic with an unadjusted cosine kernel matrix, when the gene expression was simulated using adjusted cosine kernel covariance matrices. The KS test p-values are not displayed as the distributions are visibly different.

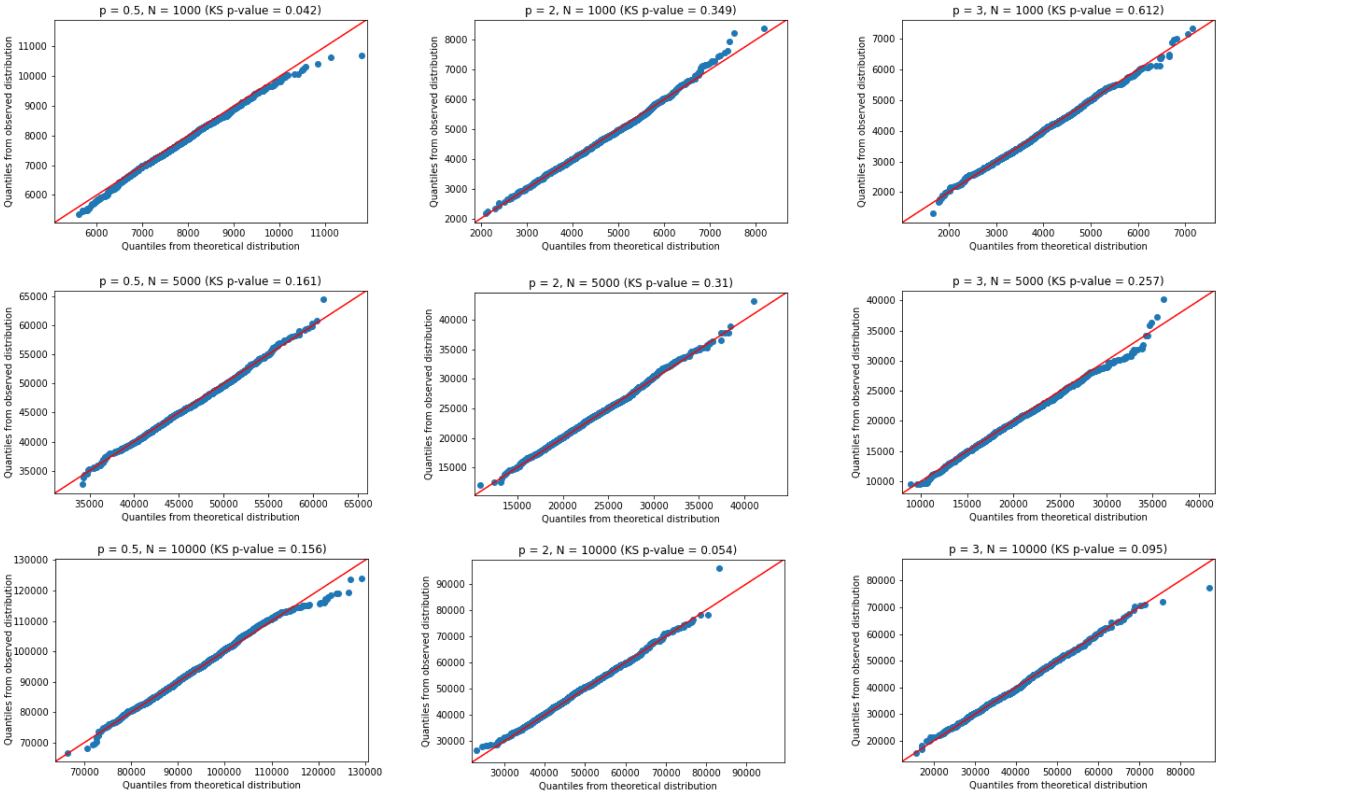

Fig D: The QQ plot of the observed and theoretical distribution of the test statistic with an adjusted cosine kernel matrix. The gene expression was simulated using adjusted cosine kernel covariance matrices. The KS test p-values are displayed in the title of every sub-figure.

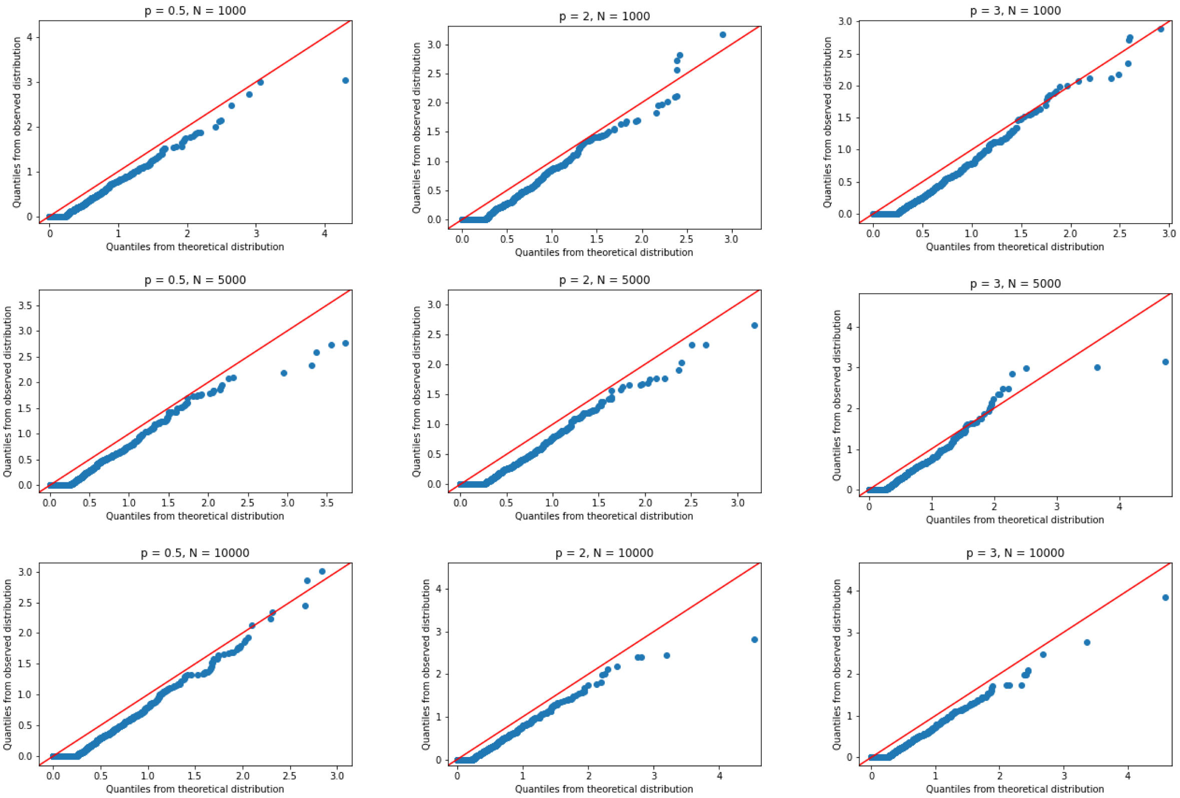

Fig E: The QQ plot of the observed and theoretical distribution of the $-\log_{10}(p)$-values. The gene expression was simulated using adjusted cosine kernel covariance matrices.

References

[1] Dennis D Boos, Leonard A Stefanski, et al. *Essential statistical inference.* Springer, 2013.

[2] Yuhan Hao, Stephanie Hao, Erica Andersen-Nissen, William M Mauck, Shiwei Zheng, Andrew Butler, Maddie J Lee, Aaron J Wilk, Charlotte Darby, Michael Zager, et al. Integrated analysis of multimodal single-cell data. *Cell*, 184(13):3573–3587, 2021.

[3] Yaowu Liu, Sixing Chen, Zilin Li, Alanna C Morrison, Eric Boerwinkle, and Xihong Lin. Acat: a fast and powerful p value combination method for rare-variant analysis in sequencing studies. *The American Journal of Human Genetics*, 104(3):410–421, 2019.

[4] Shawn R Narum. Beyond bonferroni: less conservative analyses for conservation genetics. *Conservation genetics*, 7:783–787, 2006.

[5] Shiquan Sun, Jiaqiang Zhu, and Xiang Zhou. Statistical analysis of spatial expression patterns for spatially resolved transcriptomic studies. *Nature methods*, 17(2):193–200, 2020.

[6] Valentine Svensson, Sarah A Teichmann, and Oliver Stegle. Spatialde: identification of spatially variable genes. *Nature methods*, 15(5):343–346, 2018.

[7] Lukas M Weber, Arkajyoti Saha, Abhirup Datta, Kasper D Hansen, and Stephanie C Hicks. nnsvg for the scalable identification of spatially variable genes using nearest-neighbor gaussian processes. *Nature Communications*, 14(1):4059, 2023.

[8] Jiaqiang Zhu, Shiquan Sun, and Xiang Zhou. Spark-x: non-parametric modeling enables scalable and robust detection of spatial expression patterns for large spatial transcriptomic studies. *Genome Biology*, 22(1):1–25, 2021.
