# Supplementary material for: SMASH: Scalable Method for Analyzing Spatial Heterogeneity of genes in spatial transcriptomics data
Source: PLoS Genet. 2023 Oct 20;19(10):e1010983. doi: 10.1371/journal.pgen.1010983 (PMC10619839; doi:10.1371/journal.pgen.1010983)
Supplement: Attachment — Submitted filename: 20230512_plos_genetics.pdf [file pgen.1010983.s002.pdf]

Report on

SMASH: Scalable method for analyzing spatial heterogeneity of genes in spatial transcriptomics data

This paper proposes a non-parametric computationally scalable metho to detect spatially variable genes in spatial transcriptomic data. I have the following questions for the authors to consider:

1. The proposed method is very similar to SPARK-X where a kernel calculates the locations. A natural question arises: can you use the kernel to calculate the expression level and compute $tr(H(E_k)S)/N$?

2. It would be very helpful for the authors to provide intuition on why the proposed method is more powerful. Is it because that using a kernel on the locations makes it easier to detect non-linear relationships?

3. I did not quite get why to use the minimum p-value combination rule. The minimum p value seems to be for meta-analysis. Different kernels give the method power for different spatial cluster patterns. Is it more suitable to use the Cauchy combination rule as in SPARK?

4. Can the authors provide a table contrasting computational time using different methods?
