# Supplementary material for: SMASH: Scalable Method for Analyzing Spatial Heterogeneity of genes in spatial transcriptomics data
Source: PLoS Genet. 2023 Oct 20;19(10):e1010983. doi: 10.1371/journal.pgen.1010983 (PMC10619839; doi:10.1371/journal.pgen.1010983)
Supplement: Attachment — Submitted filename: SMASH_responses.pdf [file pgen.1010983.s003.pdf]

Response to the comments for "SMASH: Scalable Method for Analyzing Spatial Heterogeneity of genes in spatial transcriptomics data"

Souvik Seal [1*], Benjamin G. Bitler[2], and Debashis Ghosh [31]

[1]Department of Public Health Sciences, School of Medicine, Medical University of South Carolina, Charleston, USA

[2]Department of Obstetrics and Gynecology, School of Medicine, University of Colorado Denver Anschutz Medical Campus, Aurora, USA

[3]Department of Biostatistics and Informatics, Colorado School of Public Health, University of Colorado Denver Anschutz Medical Campus, Aurora, USA

General comments for the reviewers:

1. We would like to thank all the reviewers and the editor for their comments, which we have addressed in detail in the following sections. During the initial submission on the PLOS Genetics portal, we uploaded the .tex file of the manuscript and the high-quality tiff versions of the figures, separately. However, when the portal compiled the .tex file and figures together, the figures appeared to be very low-resolution in the resulting PDF. We contacted the journal

office which said that the links to the original versions of the figures would appear in the compiled PDF. This time, we are attaching the figures in a separate PDF file, titled "Figures of the main text", to the portal just to make sure that the reviewers can easily access them. We hope that the figures stay legible, and apologize for the poor-quality images from the last submission.

2. The notation $H(S)$, previously used to denote the kernel covariance matrix for location variable S, has now been replaced with $K_{\mathcal{S}}$ in every text.

Reviewer 1

Major comments:

1. *"The proposed method is very similar to SPARK-X where a kernel calculates the locations. A natural question arises: can you use the kernel to calculate the expression level and compute tr(H(Ek)S)/N?"*

Response: We thank the reviewer for the thoughtful comment. The proposed test statistic has the form, $T_k^{\text{SMASH}} \equiv tr(E_k K_{\mathcal{S}})/N$, where $E_k = y_k(y_k^T y_k)^{-1} y_k^T$ and $K_{\mathcal{S}}$ can have different structures based on the choice of the kernel covariance function, such as Gaussian, cosine, and linear. We agree that the test statistic is similar to SPARK-X but it is more general for the reasons iterated in the reply to the reviewer's second comment below. If we understand correctly, the reviewer is suggesting the possibility of computing the kernel covariance matrix based on y_k instead of S. Ideally, to achieve the highest power, one should construct kernel covariance matrices based on both y_k and S. It would, however, increase the computational burden. First, recall that to compute $tr(AB)$ for two square matrices, $A = [[a_{ij}]]_{N\times N}$ and $B = [[b_{ij}]]_{N\times N}$, we require a computational

complexity of $O(N^2)$, using the property that $tr(AB) = \sum_{i=1}^{N} \sum_{j=1}^{N} a_{ij} b_{ij}$ which needs computing element-wise product of the two $N \times N$ matrices, i.e., performing N^2 multiplications, and summing up those values [29]. Now, let us focus on the expression of T_k^{SMASH} and its asymptotic null distribution. By keeping E_k to have a single form and considering multiple choices (say, R_1) of $K_{\mathcal{S}}$, we require to compute R_1 different values of $tr(E_k K_{\mathcal{S}})$, with each computation demanding $O(N^2)$ complexity. Additionally, the asymptotic null distribution of T_k^{SMASH} requires computing the variance: $\mathbb{V}(T_k^{\text{SMASH}}) = \frac{2}{N^4} tr(E_k^2) tr(K_{\mathcal{S}}^2)$. Note that $tr(E_k^2) = 1$, when $E_k = y_k(y_k^T y_k)^{-1} y_k^T$. But, when E_k does not have such a projection matrix-like structure, we would also need to compute $tr(E_k^2)$. Thus, if we consider R_2 different choices of E_k instead of keeping it fixed, we will need to compute $(R_1 \times R_2)$ different values of $tr(E_k K_{\mathcal{S}})$ and also, R_2 different values of $tr(E_k^2)$. In total, we will thus require $(R_1 \times R_2 + R_2 - R_1)$ additional computations, each of $O(N^2)$ complexity.

To understand why considering multiple choices of E_k might be more powerful, we need to discuss the theoretical background of the proposed test. We are testing the null hypothesis of y_k and S being independent, i.e., $H_0 : y_k \perp\!\!\!\perp S$, using a non-parametric kernel-based framework [8, 10, 9, 11]. To define the test formally, let y_k and S have domains $\mathcal{Y}$ and $\mathcal{S}$, respectively. Denote $k_{\mathcal{Y}}$ and $k_{\mathcal{S}}$ to be two measurable positive definite (PD) kernels with the corresponding reproducible kernel Hilbert spaces (RKHSs) denoted by $H_{\mathcal{Y}}$ and $H_{\mathcal{S}}$ on $\mathcal{Y}$ and $\mathcal{S}$, respectively. Then, the cross-covariance operator: $\Sigma_{\mathcal{S}\mathcal{Y}}$ from $H_{\mathcal{Y}}$ to $H_{\mathcal{S}}$ can be defined by the relation: $< f_1, \Sigma_{\mathcal{S}\mathcal{Y}} f_2 >_{H_{\mathcal{S}}} = cov(f_2(y_k), f_1(S)), \forall f_1 \in H_{\mathcal{S}}$, $f_2 \in H_{\mathcal{Y}}$, where $< . >$ denotes an inner product. $\Sigma_{\mathcal{S}\mathcal{Y}}$ can be interpreted as a more general version of the covariance matrix on Euclidean spaces, representing higher-order correlations of y_k and S through $f_2(y_k)$ and $f_1(S)$. Under additional regulatory assumptions on RKHSs: $H_{\mathcal{Y}}$ and $H_{\mathcal{S}}$, it can be shown that testing $H_0 : y_k \perp\!\!\!\perp S$ is equivalent to testing, $H_0 : \Sigma_{\mathcal{S}\mathcal{Y}} = 0$. This testing can be performed using a test statistic of the form $tr(K_{\mathcal{Y}} K_{\mathcal{S}})$, where $K_{\mathcal{Y}}$ and $K_{\mathcal{S}}$ are the kernel covariance or Gram matrices, obtained using the PD kernels: $k_{\mathcal{Y}}$ and $k_{\mathcal{S}}$. Notice that T_k^{SMASH} has

a similar structure as well. In the context of real-world problems, exact choices for $k_{\mathcal{Y}}$ and $k_{\mathcal{S}}$ are never known, which is why one considers different options and aggregates the results. In our case, we are considering a single kernel (linear kernel) choice for $k_{\mathcal{Y}}$ that gives us the Gram matrix $K_{\mathcal{Y}} = E_k = y_k(y_k^T y_k)y_k^T$, while for $k_{\mathcal{S}}$ we consider multiple options, leading to different forms of the Gram matrix $K_{\mathcal{S}}$. If one decides to vary the choice of $k_{\mathcal{Y}}$ as well, the test statistic could potentially become more powerful since it would enable the detection of newer non-linear patterns between y_k and S. However, as pointed out earlier, considering these many different kernel combinations will not only be more computationally demanding but also may turn out to be redundant, with no significant gain in power. This issue of choosing an optimal set of kernel matrices was partly studied in the statistical genetics literature [37], and it will be an important topic for future research to study how it translates in the context of ST datasets.

2. *"It would be very helpful for the authors to provide intuition on why the proposed method is more powerful. Is it because that using a kernel on the locations makes it easier to detect non-linear relationships?"*

Response: We thank the reviewer for the comment. The gain in power can be partially justified using the argument provided in the reply to the reviewer's first comment. Recall that we have two kernels: $k_{\mathcal{Y}}$ and $k_{\mathcal{S}}$ of which the first one is kept fixed to be a linear kernel while the latter is varied in both the methods. In SPARK-X, Zhu et al. (2021) [40] considers $k_{\mathcal{S}}$ to be linear but transforms S to $g(S)$ where g can be identity, Gaussian and cosine transformation. It leads to a very specific projection matrix-like form of the Gram matrix: $K_{\mathcal{S}} = g(S)(g(S)^T g(S))^{-1} g(S)^T$. Such a simplistic structure of $K_{\mathcal{S}}$, however, only permits capturing linear dependence between y_k and $g(S)$. In SMASH, we consider kernel $k_{\mathcal{S}}$ to be not only linear (i.e., $K_{\mathcal{S}} = g(S)(g(S)^T g(S))^{-1} g(S)^T$) but also Gaussian

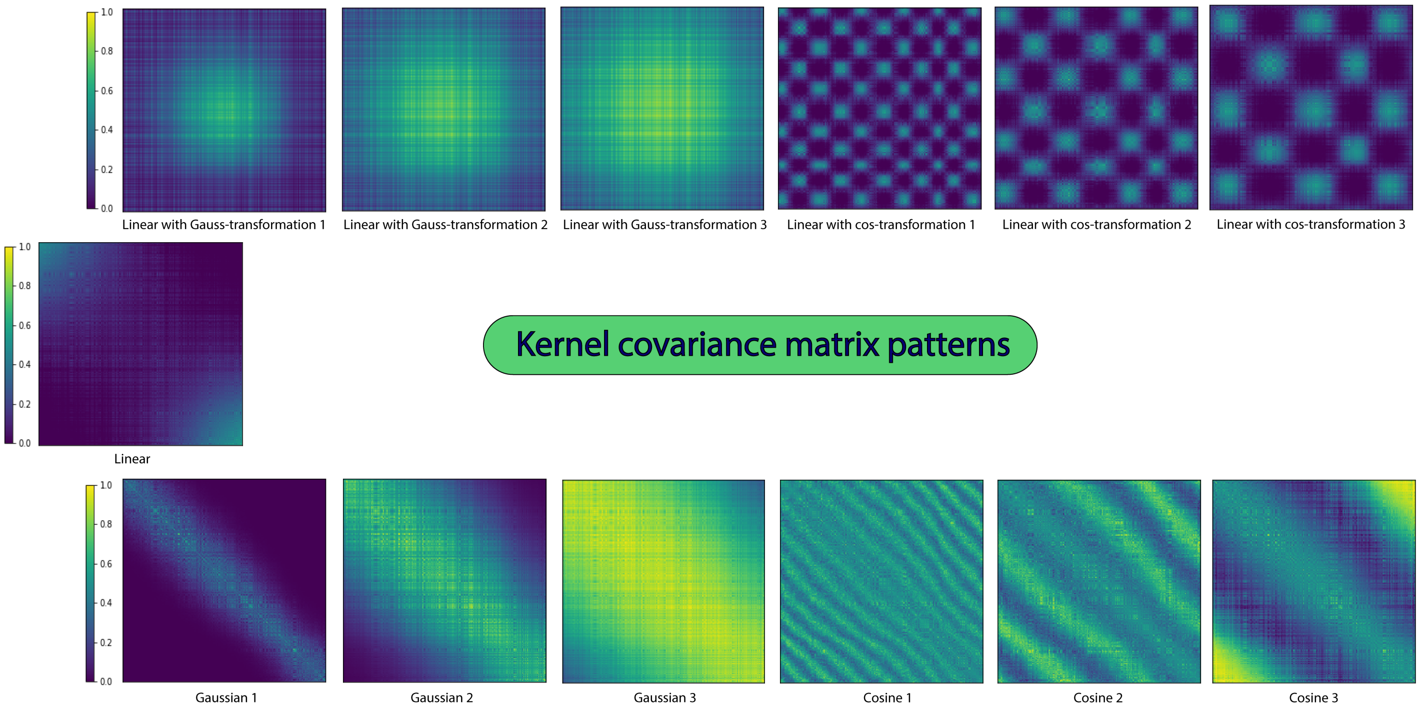

Figure 1: The patterns of kernel covariance matrices for different choices of kernels, namely, linear, Gaussian, Cosine, linear with Gaussian-transformed coordinates, and linear with cosine-transformed coordinates. Three values of the associated hyper-parameter have been considered for every kernel (except linear), e.g., Gaussian 1, Gaussian 2, and Gaussian 3 correspond to three different values (from low to high) of the length-scale parameter l, respectively. SMASH is capable of capturing all of these patterns in the gene expression, while SPARK-X captures only the patterns in the top row and the one in the middle.

and cosine, leading to more general forms of $K_{\mathcal{S}}$, such as $K_{\mathcal{S}} = \left[\left[\cos\left(\frac{2\pi||s_i - s_j||}{p}\right)\right]\right]_{N\times N}$; $||s_i - s_j||= \sqrt{(s_{i1}-s_{j1})^2 + (s_{i2}-s_{j2})^2}$. It enables us to capture more non-linear patterns of dependence between y_k and S, resulting in more power. We have added this figure (Figure 1 of this text) to the supplementary information, which shows different spatial patterns our method is capable of capturing.

3. *"I did not quite get why to use the minimum p-value combination rule. The minimum p value seems to be for meta-analysis. Different kernels give the method power for different spatial cluster patterns. Is it more suitable to use the Cauchy combination rule as in SPARK?"*

Response: We thank the reviewer for the comment. We agree that the minimum p-value combination rule is one of the most basic ways of combining multiple p-values. The primary reason behind considering this rule was its simple formula and familiar interpretation. In our simulation results, the Cauchy rule [19] would sometime produce inflated type-1 error, while the minimum p-value rule would not exhibit any such problem. Based on the reviewer's comment, we have now added the functionality into our *Python* package to choose one of these two combination rules.

4. *"Can the authors provide a table contrasting computational time using different methods?"*

Response: We thank the reviewer for the comment. In Table 1 of the main text, we have provided the computational complexity of different algorithms and their actual run times (in seconds) for varying values of the number of locations N.

Table 1: **Computational complexity and run-time comparison.** The table lists the theoretical complexity and run-time (in seconds) of the four methods, SMASH, SPARK-X, SpaGene, and SpatialDE in a simulation setup with $K = 1000$ genes and varying number of cells N. The number of spatial co-ordinates d was equal to 2. **SpaGene constructs multiple kNN graphs and performs permutation tests. We are only listing the complexity of the KNN algorithm.*

Method	Complexity	N = 1000	N = 5000	N = 10000
SMASH	$O(N^2K)$	0.9	18.5	97.5
SPARK-X	$O(NKd^2)$	0.72	4.1	4.3
SpaGene*	$O(NKd)^*$	0.2	1.1	2.1
SpatialDE	$O(N^3 + N^2K)$	24.5	245	971.7

Reviewer 2

Major comments:

1. *"Even at the beginning of the introduction, the authors made an inappropriate statement. Some widely used sequencing-based spatially resolved transcriptomics (SRT) technologies, such as 10x Visium cannot achieve a much finer spatial resolution than those imaging-based SRT technologies. The authors need to do more research in this field, and know the strengths of different platforms."*

Response: We thank the reviewer for the comment. We apologize for listing "Visium as operating on a much finer spatial resolution" and also for not clearly distinguishing between different ST technologies and relevant platforms. We have now added the following paragraph to the introduction section of the main manuscript,

The popular ST technologies and corresponding platforms differ in terms of the procedure used to

record spatial profiles, such as region of interest (ROI) selection [42, 22], next-generation sequencing (NGS) with spatial barcoding [27, 30, 34], and single-molecule fluorescence in situ hybridization (smFISH) [16, 20, 4]. Two crucial aspects that a researcher considers before choosing a suitable platform, are a) the capability of transcriptome-wide profiling, and b) the granularity of spatial resolution. For example, the majority of the smFISH-based technologies excel at capturing single-cell level resolution but lack the capability of transcriptome-wide profiling. On the other hand, ROI or NGS-based technologies can be used for transcriptome-wide profiling but on a significantly lower spatial resolution, such as 55 μm for the most popular and commercialized ST platform Visium (10X Genomics). We refer to Moses et al. (2022) [24] for a detailed discussion on these technologies. Deriving biological insights from datasets obtained using such platforms with either huge spatial or genomic profiles, or both, not only poses numerous statistical challenges but also requires maximum computational efficiency [1].

2. *"There are many more SVG detection methods that have been recently developed. However, the authors ignore them. A more comprehensive literature survey is required."*

Response: We thank the reviewer for the great comment. We have now added a more complete discussion regarding the available methods as follows,

The methods can be broadly classified into three types: a) based on statistical modeling, b) based on machine learning or neural network, and c) based on graphical networks or spatial grids. Some of the notable methods of each type would be, type (a): Trendsceek [7], SpatialDE [33], SPARK [32], SPARK-X [40], and Boost-GP [17], type (b): SPADE [2], SOMDE [12], and SpaGCN [13], and type (c): HMRF [41], MERINGUE [23], Binspect-Giotto [6], Boost-MI [14], ScGCO [38], and SpaGene [18].

3. *"Although much details were provided in the "A brief overview of existing methods" section, the authors still used two page on explaining SpatialDE, SPARK, SPARK-X, and SpaGene, which is not necessary. Instead, the authors just need to briefly present their ideas, pros, and cons, especially in terms of time complexity (like Table 1). The derivation of complexity needs to be fully explained or cited."*

Response: We thank the reviewer for the comment. Some parts of the discussion are required to explain the computational complexity of the existing methods. However, we do agree that there were some unnecessary details which we have now removed, making the discussion more succinct. Following the reviewer's suggestion we have now added Table 2 listing the kernels considered by different methods. The complexity mainly centers around computing the trace of the product of two

Table 2: **Kernel choices in different methods.** The table shows (yes/no) if a particular kernel covariance or Gram matrix is considered in different methods.

Method	Linear	Gaussian	Cosine	Linear with transformed coordinates
SMASH	Yes	Yes	Yes	Yes
SPARK-X	Yes	No	No	Yes
SpatialDE	Yes	Yes	Yes	No
SPARK-X	Yes	Yes	Yes	No

square matrices. To compute $tr(AB)$ for two square matrices, $A = [[a_{ij}]]_{N\times N}$ and $B = [[b_{ij}]]_{N\times N}$, we use the property that $tr(AB) = \sum_{i=1}^{N}\sum_{j=1}^{N} a_{ij}b_{ij}$ which requires a complexity of $O(N^2)$, since we are performing element-wise product of the two $N \times N$ matrices, i.e., performing N^2 multiplications, and summing up those values. We have added this reference: Skienna (1998) [29]. We have also discussed our test in more detail and clarity. Below are the changes incorporated in the main text,

We test the null hypothesis of y_k and S being independent, i.e., $H_0 : y_k \perp\!\!\!\perp S$, using a non-parametric kernel-based framework [8, 10, 9, 11]. Let y_k and S have domains $\mathcal{Y}$ and $\mathcal{S}$, respectively. Denote $k_{\mathcal{Y}}$ and $k_{\mathcal{S}}$ to be two measurable positive definite (PD) kernels with the corre-

sponding reproducible kernel Hilbert spaces (RKHSs) denoted by $H_{\mathcal{Y}}$ and $H_{\mathcal{S}}$ on $\mathcal{Y}$ and $\mathcal{S}$, respectively. Then, the cross-covariance operator: $\Sigma_{\mathcal{S}\mathcal{Y}}$ from $H_{\mathcal{Y}}$ to $H_{\mathcal{S}}$ can be defined by the relation: $< f_1, \Sigma_{\mathcal{S}\mathcal{Y}} f_2 >_{H_{\mathcal{S}}} = cov(f_2(y_k), f_1(S)), \forall f_1 \in H_{\mathcal{S}},\ f_2 \in H_{\mathcal{Y}}$, where $< . >$ denotes an inner product. $\Sigma_{\mathcal{S}\mathcal{Y}}$ can be interpreted as a more general version of the covariance matrix on Euclidean spaces, representing higher-order correlations of y_k and S through $f_2(y_k)$ and $f_1(S)$. Under additional regulatory assumptions on RKHSs: $H_{\mathcal{Y}}$ and $H_{\mathcal{S}}$, it can be shown that testing $H_0 : y_k \perp\!\!\!\perp S$ is equivalent to testing, $H_0 : \Sigma_{\mathcal{S}\mathcal{Y}} = 0$. This testing can be performed using a test statistic of the form $tr(K_{\mathcal{Y}} K_{\mathcal{S}})$, where $K_{\mathcal{Y}}$ and $K_{\mathcal{S}}$ are the kernel covariance or Gram matrices, obtained using the PD kernels: $k_{\mathcal{Y}}$ and $k_{\mathcal{S}}$ [25, 5]. In the context of real datasets, exact choices for $k_{\mathcal{Y}}$ and $k_{\mathcal{S}}$ are never known. Therefore, we consider different kernel choices and aggregate the results. Our test statistic has the form $T_k^{\text{SMASH}} \equiv tr(E_k K_{\mathcal{S}})/N$, where E_k is defined as earlier, i.e., $k_{\mathcal{Y}}$ is fixed to be a linear kernel, while $k_{\mathcal{S}}$ and consequently, $K_{\mathcal{S}}$ is varied to have different forms as described next.

For the Gaussian and cosine covariance matrices, we consider ten data-driven fixed values of the lengthscale l and period p, respectively. For further details, refer to the supplementary information. Refer to Figure S1 in the supplementary information to visualize the spatial patterns corresponding to the different kernel covariance matrices. In Table 2, we list the kernel covariance matrices used in different methods. Note that T_k^{SPARKX} can be interpreted as a special case of T_k^{SMASH} as the former only considers linear kernel covariance matrices.

However, unlike the kernel choices of T_k^{SPARKX}, $K_{\mathcal{S}}$ does not always have a projection matrix-like structure of D, and thus, its eigenvalues can not be computed with the complexity of $O(Nd^2)$. Instead, it requires the complexity of $O(N^3)$, which becomes intractable as N increases. Therefore, we consider a variation of Welch-Satterthwaite approximation [36, 28], to approximate the asymptotic null distribution of T_k^{SMASH} with a gamma distribution [11] as below

4. *"All figures are in super-low quality. They are too blur to get any messages. In Figure 1, it is observed SMASH has a marginal improvement than SPARK-X. The visualization of the simulation results needs to be improved. I don't think line plot is a good choice under this circumstance."*

Response: We thank the reviewer for the comment. As we have mentioned before, the figures became low quality after compilation in the PLOS Genetics portal. We have contacted the journal office which said that the links to the original high-quality figures will be provided at the top of every page. We have now provided the figures in a separate PDF file. The clear difference between the power of SMASH and SPARK-X is visible in Figures 2 and 3 (of the main text) which correspond to the simulation setups 2 and 3, respectively, of the main text, both based on the Gaussian process (GP)-based spatial regression model from SpatialDE [33].

5. *"SMASH is a natural extension of SPARK-X, where the distance covariance matrix D in the test statistics, T=tr(E_k D)/N has been replaced by kernel-based distance covariance matrix. Thus, the contribution is very minimal in the field."*

Response: We thank the reviewer for the comment. We do agree that our test is a natural extension of SPARK-X as it augments the latter by incorporating additional kernel covariance matrices, namely Gaussian and cosine with the forms $K_{\mathcal{S}} = \left[\left[\exp\left(-\frac{||s_i - s_j||^2}{2l^2}\right)\right]\right]_{N\times N}$, and $K_{\mathcal{S}} = \left[\left[\cos\left(\frac{2\pi||s_i - s_j||}{p}\right)\right]\right]_{N\times N}$, respectively, where $||s_i - s_j|| = \sqrt{(s_{i1} - s_{j1})^2 + (s_{i2} - s_{j2})^2}$. For a visual representation of the new patterns SMASH can detect by considering these additional kernel matrices, refer to Figure 1 (of this file), and the reply to reviewer 1's comment 2. To reiterate, SPARK-X only captures a linear or first-order dependency between the gene expression y_k and transformed location S: $g(S)$, i.e., SPARK-X is approximately equivalent to fitting a simple linear model: $y_k = g(S)\beta_k + \epsilon_k$ and testing if the fixed effect β_k differs from 0, where g can be either identity, Gaussian, or cosine transformation (not to be confused with the kernel matrices). However,

older methods like SpatialDE [33] and SPARK [32], are capable of detecting higher-order dependencies between y_k and S by assuming that the covariance of y_k is proportional to the Gaussian and cosine kernel covariance matrices. However, being reliant on an explicit estimation of variance parameters, these methods lack computational feasibility as the number of cells/spots N increases. In the proposed method, our goal is to harness the power of these older methods, while staying computationally as feasible as possible. In addition to that, a key aim of the paper is to demonstrate through extensive simulations and real data analyses that a method like SPARK-X, although highly scalable due to its simplistic assumptions, can suffer profusely in terms of SVG detection power, necessitating the use of more sophisticated alternatives.

6. *"The authors directly use the results "the asymptotic null distribution of T_k can be well approximated by a gamma distribution" without validating it in the simulation study. Actually, the results were from an unpublished paper on arXiv 10 years ago. I doubt if the approximation is valid in the context of SVG detection, especially when N is large. Zhang K, Peters J, Janzing D, Sch ¨¨olkopf B. Kernel-based conditional independence test and application in causal discovery. arXiv preprint arXiv:12023775. 2012;."*

Response: We thank the reviewer for the great comment. The testing framework we use has previously been discussed in many published works in different forms [8, 10, 9, 11] which we apologize for not listing clearly. The test is also popularly known as the Hilbert-Schmidt independence criterion or HSIC. As $N \to \infty$, the statistic has been shown to follow a weighted mixture of independent χ_1^2 distributions under the null hypothesis, which can be represented in two forms, one outlined in [39] and a slightly complicated one derived in [11]. Now, both of these mixture χ^2 distributions can be approximated using a two-parameter Gamma distribution using a variation of the Welch-Satterthwaite approximation [28, 36, 15, 10]. These two parameters of the Gamma

distribution can be estimated by computing the empirical mean and variance of the test statistic, for a complete derivation of which, we refer to Gretton et al. (2007) [11] (manuscript available at this link, http://www.gatsby.ucl.ac.uk/ gretton/papers/GreFukTeoSonetal08.pdf). We are using these same formulae in the manuscript and our developed package.

Following the reviewer's suggestion, we investigated how well the approximate distribution performs in the context of SVG detection. Recall the form of our test statistic, $T_k^{\text{SMASH}} = tr(E_k K_{\mathcal{S}})/N$. First, we generated data under the null hypothesis using the simulation setup (2) of the main text which uses the Gaussian process (GP)-based spatial regression model from SpatialDE [33] with Gaussian kernel covariance matrices. In Figure 2, we show the quantile-quantile (QQ) plot of the observed distribution (on the y-axis) and the theoretical distribution (on the x-axis) of the test statistic T_k^{SMASH} with $K_{\mathcal{S}}$ being the Gaussian kernel matrix, along with the p-values from a Kolmogorov-Smirnov (KS) test [21]. Notice that the distributions visibly agreed with each other in every case, which was further supported by the KS p-values being ≥ 0.05. Next, we generated data under the null hypothesis using the same Gaussian process (GP)-based spatial regression model [33], with cosine or periodic kernel covariance matrices. A problem with considering a cosine kernel covariance matrix is its lack of positive-definiteness. In our simulation model, we adjusted the cosine kernel covariance matrix by forcing the negative eigenvalues to be equal to 0, a strategy that is used in both SpatialDE and SPARK models [33, 32]. A crucial assumption behind our test, as described in the reply to reviewer 1's comment 2, is that the kernels $k_{\mathcal{Y}}$, $k_{\mathcal{S}}$ and consequently, the corresponding covariance or Gram matrices need to be positive-definite. Thus, the distribution we have been considering, only holds if $K_{\mathcal{S}}$ is positive-definite. In Figures 3 and 4, we display the QQ plots of the empirical and theoretical distribution of our test statistic with unadjusted and adjusted (i.e., truncating negative eigenvalues to be 0) cosine kernel covariance matrices, respectively. By adjustment we mean replacing $K_{\mathcal{S}} = \sum_{i=1}^{N} \lambda_i U_i U_i^T$ by $K_{\mathcal{S}}^* = \sum_{i=1}^{N} \max\{\lambda_i, 0\} U_i U_i^T$, where λ_i and

U_i denote the i-th eigenvalue and eigenvector, respectively. Quite expectedly, with an unadjusted cosine kernel matrix, the empirical distribution differed significantly from the theoretical distribution but the extreme values were below the $y = x$ line, indicating a conservative nature of the test. With an adjusted cosine kernel matrix, the distributions mostly agreed with each other. Keep in mind that to adjust a cosine or periodic kernel matrix, we first need to compute its eigenvalues, a step requiring computational complexity of $O(N^3)$. Unlike traditional methods like SpatialDE and SPARK-X, we do not want to follow this route to avoid the computational burden. Instead, we argue that the simple unadjusted test statistic is conservative in nature and thus, can be usable in most practical scenarios. In Figure 5, we show the QQ plot of the observed and theoretical distributions of $-\log_{10}(p)$-values obtained by our aggregate test (i.e., p-values were combined for different kernel choices) in the same simulation setup. Notice that the test was conservative in all cases. In null simulations using all four real datasets as well, we noticed similar QQ plots which are provided in the supplementary information. Another simplistic solution could be to drop the cosine kernels entirely from the analysis, following methods like SOMDE [12] and nnSVG [35]. We have updated our software now to provide users to choose one of the three options: a) consider unadjusted cosine kernels, b) consider adjusted cosine kernels, and c) do not consider cosine kernels. We have added these comments and plots to the supplementary information, and also incorporated this discussion briefly into the main manuscript as,

The cosine or periodic kernel covariance matrix is not positive definite (PD). Our testing framework and the distributional derivations hold only for PD kernel covariance matrices. One solution could be to truncate the negative eigenvalues of the kernel matrix, i.e., adjusting $K_{\mathcal{S}} = \sum_{i=1}^{N} \lambda_i U_i U_i^T$ as $K_{\mathcal{S}}^* = \sum_{i=1}^{N} \max\{\lambda_i, 0\} U_i U_i^T$, where λ_i and U_i denote the i-th eigenvalue and eigenvector, respectively. However, computing eigenvalues can become computationally challenging as it requires a complexity of $O(N^3)$. In our simulations, we have noticed that using unadjusted versions of the

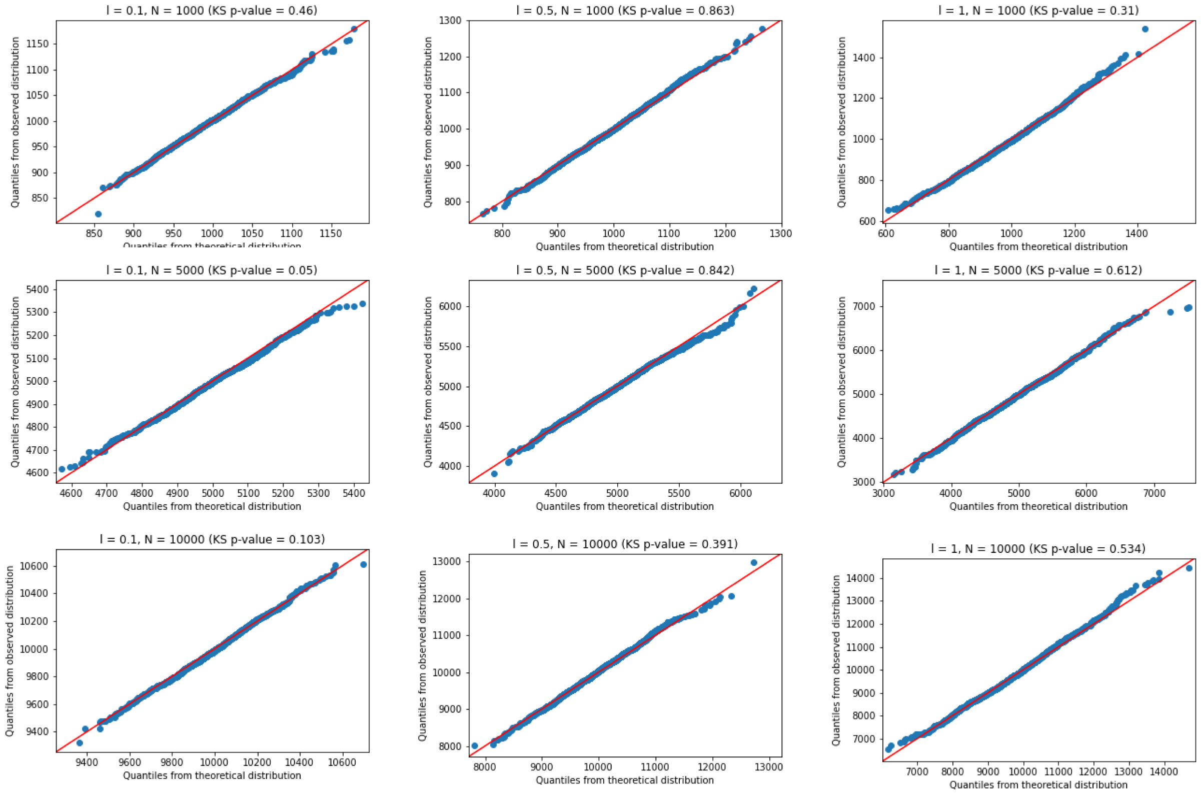

Figure 2: The QQ plot of the observed and theoretical distribution of the test statistic, when the gene expression was simulated using Gaussian kernel covariance matrices. The KS test p-values are displayed in the title of every figure.

kernel matrices yielded conservative test results, with no sign of p-value inflation. We refer to the supplementary information for more details.

7. *"Lack of details about how to specify the data-driven fixed values of the length-scale characteristic parameter l, which has a huge impact on the kernel-based covariance matrix."*

Response: We thank the reviewer for the comment. We follow a strategy similar to both SpatialDE [33] and SPARK [32] to vary the lengthscale parameter l and the period parameter p from low to high values. To elaborate, we use ten Gaussian kernels with pre-fixed l values together with ten cosine kernels with pre-fixed p values (not to be confused with p-value). The ten choices of l and p

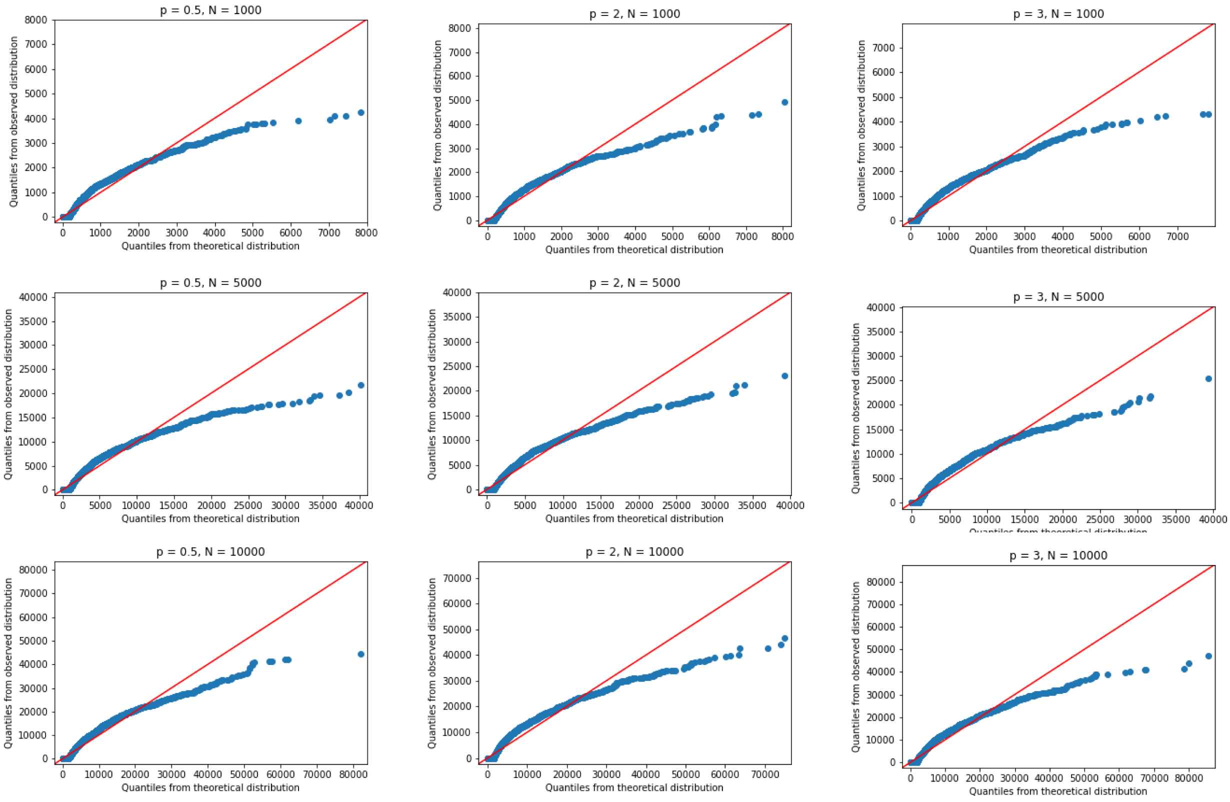

Figure 3: The QQ plot of the observed and theoretical distribution of the test statistic with an unadjusted cosine kernel matrix, when the gene expression was simulated using adjusted cosine kernel covariance matrices. The KS test p-values are not displayed as the distributions are visibly different.

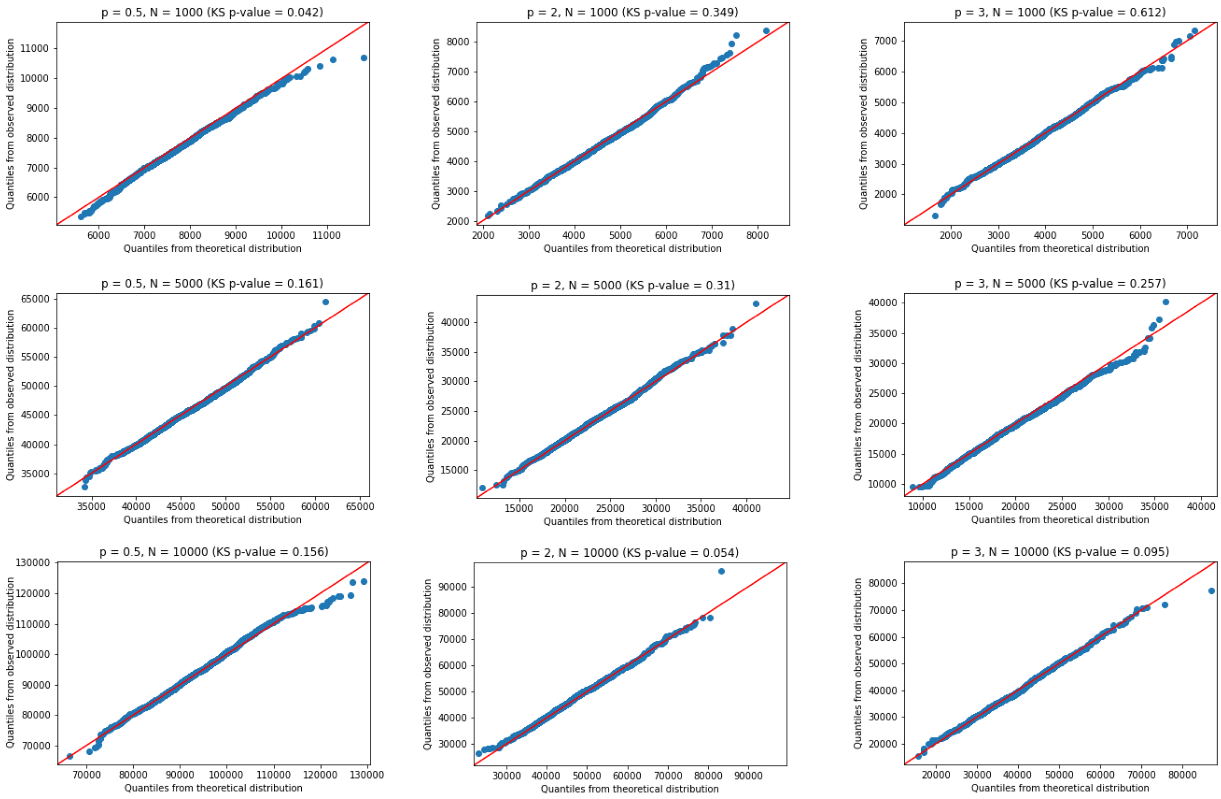

Figure 4: The QQ plot of the observed and theoretical distribution of the test statistic with an adjusted cosine kernel matrix. The gene expression was simulated using adjusted cosine kernel covariance matrices. The KS test p-values are displayed in the title of every figure.

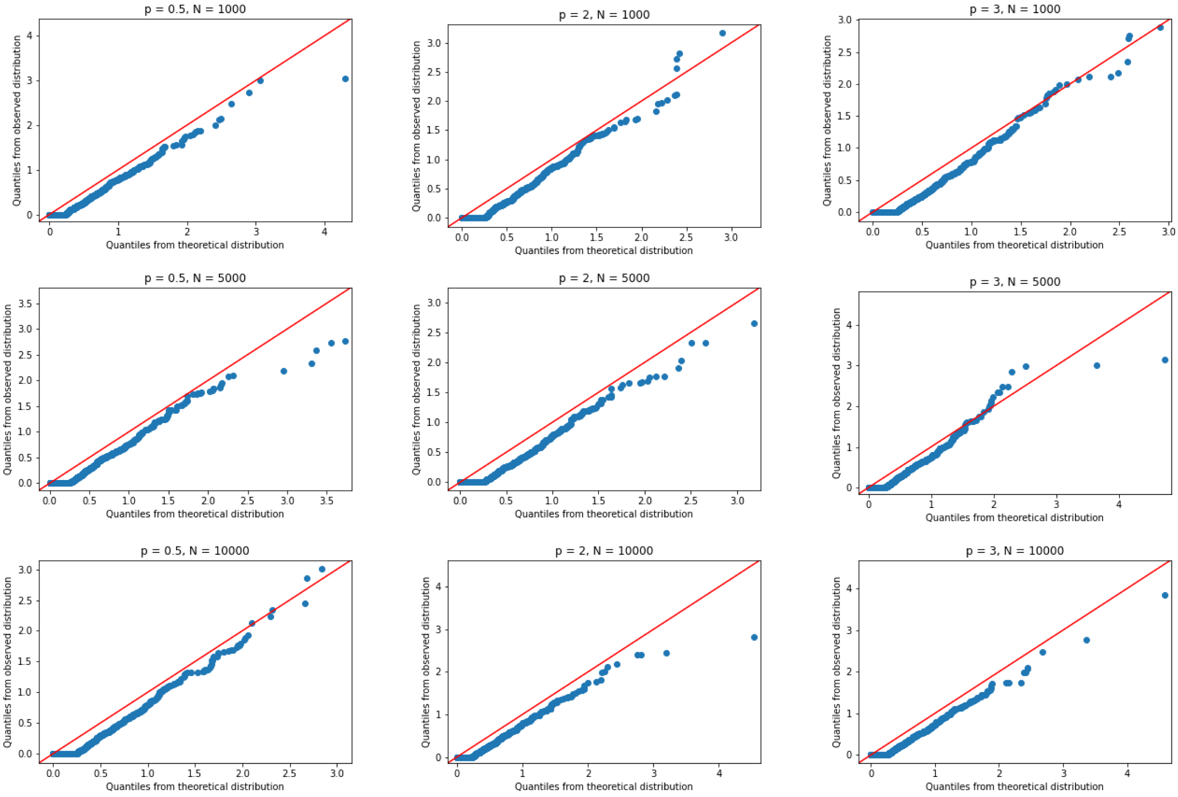

Figure 5: The QQ plot of the observed and theoretical distribution of the $-\log_{10}(p)$-values. The gene expression was simulated using adjusted cosine kernel covariance matrices.

are determined based on the pair-wise Euclidean distances among spatial locations in the data, i.e., $||s_i - s_j||= \sqrt{(s_{i1} - s_{j1})^2 + (s_{i2} - s_{j2})^2}$ for $(i, j) \in \{1, 2, \ldots, N\}$. We compute the minimum (say, d_{min}) and maximum (say, d_{max}) values of the non-zero Euclidean distances across all pairs of spatial locations. Then, we chose ten equally spaced values between $(\log_{10}(d_{min}/2), \log_{10}(2d_{max}))$. Finally, we take power ten of the values to bring back to the original scale of distances and consider them as values of l and p. Note that such a kind of grid selection for the hyperparameters was originally discussed in [26].

8. *"The real data analyses were poorly presented, and were not persuasive why the method is needed."*

Response: We thank the reviewer for the comment. We are not entirely sure if the poor presentation was due to the images appearing to be of poor quality or the analysis strategy. In the first three real data analyses, we highlighted three key things,

1. In the Venn diagrams, we showed that SMASH detected most of the SVGs that were detected by either SPARK-X or SpaGene. SPARK-X missed a lot of genes that were detected by SMASH and SpaGene. For example, in the analysis of the mouse cerebellum data, there were 203 genes detected by SMASH and SpaGene but not by SPARK-X. On top of that, SMASH detected 363 additional SVGs, missed by both SPARK-X and SpaGene. In the SCCOHT dataset, there were 1269 SVGs detected by SMASH and SpaGene which were missed by SPARK-X.

2. We performed functional enrichment analysis on the four sets of SVGS: a) the common genes identified by all three methods, b) the genes identified by SMASH and SpaGene but not by SPARK-X, c) the genes identified by SMASH and SPARK-X but not by SpaGene, and d) the genes identified only by SMASH. We also displayed the expression profile of a few

representative genes from these four sets of SVGs.

3. Finally, we computed enrichment scores (ES) of the three methods following a procedure outlined in Liu et al. (2022) [18]. Along with gene expression and spatial coordinates, we had access to information on spatial layers or clusters of every cell/spot, determined either using manual annotation or software like RCTD [3] and Seurat [12]. Suppose there were M spatial clusters in a dataset. The enrichment scores (ESs) of the three methods in each of those M clusters were computed as follows. The SVGs detected by the three methods were ranked from the most to the least significant, say as lists D_{SMASH}, $D_{SPARK-X}$, and $D_{SpaGene}$. Then, for every spatial cluster m, the top 50 genes that show enriched expression in that particular cluster were put to a list, say C_m for $m = 1, 2, \ldots, M$. Finally, an unweighted gene set enrichment analysis [31] was implemented to evaluate the enrichment of the gene set C_m in the high ranking of D_{SMASH}, $D_{SPARK-X}$, and $D_{SpaGene}$, for every m. A high enrichment score implied that the method was capable of identifying the most influential genes that dictated the identification of a spatial layer or cluster m. In the mouse cerebellum dataset, notice that for the Purkinje layer (PL), and molecular layer (ML), SPARK-X had noticeably lower enrichment scores than the other two methods. In the SCCOHT dataset, in clusters like 5, 6, and 11, SPARK-X performed significantly worse than SMASH. However, we do agree that in the human DLPFC dataset, the performance of SPARK-X and SMASH was pretty similar.

The fourth and final real dataset had 161 pre-selected markers (genes) of different cell types or in other words, highly variable genes. Although the majority of the genes were detected by all three methods, there were 6 SVGs missed by SPARK-X and 6 SVGs uniquely detected by SMASH. Again, we thank the editor and reviewers for their input and suggestions.

References

[1] Lyla Atta and Jean Fan. Computational challenges and opportunities in spatially resolved transcriptomic data analysis. *Nature Communications*, 12(1):1–5, 2021.

[2] Sungwoo Bae, Hongyoon Choi, and Dong Soo Lee. Discovery of molecular features underlying the morphological landscape by integrating spatial transcriptomic data with deep features of tissue images. *Nucleic acids research*, 49(10):e55–e55, 2021.

[3] Dylan M Cable, Evan Murray, Luli S Zou, Aleksandrina Goeva, Evan Z Macosko, Fei Chen, and Rafael A Irizarry. Robust decomposition of cell type mixtures in spatial transcriptomics. *Nature Biotechnology*, 40(4):517–526, 2022.

[4] Kok Hao Chen, Alistair N Boettiger, Jeffrey R Moffitt, Siyuan Wang, and Xiaowei Zhuang. Spatially resolved, highly multiplexed rna profiling in single cells. *Science*, 348(6233):aaa6090, 2015.

[5] Noel Cressie. *Statistics for spatial data*. John Wiley & Sons, 2015.

[6] Ruben Dries, Qian Zhu, Rui Dong, Chee-Huat Linus Eng, Huipeng Li, Kan Liu, Yuntian Fu, Tianxiao Zhao, Arpan Sarkar, Feng Bao, et al. Giotto: a toolbox for integrative analysis and visualization of spatial expression data. *Genome biology*, 22(1):1–31, 2021.

[7] Daniel Edsgärd, Per Johnsson, and Rickard Sandberg. Identification of spatial expression trends in single-cell gene expression data. *Nature methods*, 15(5):339–342, 2018.

[8] Kenji Fukumizu, Francis R Bach, and Michael I Jordan. Dimensionality reduction for supervised learning with reproducing kernel hilbert spaces. *Journal of Machine Learning Research*, 5(Jan):73–99, 2004.

[9] Kenji Fukumizu, Arthur Gretton, Xiaohai Sun, and Bernhard Schölkopf. Kernel measures of conditional dependence. *Advances in neural information processing systems*, 20, 2007.

[10] Arthur Gretton, Olivier Bousquet, Alex Smola, and Bernhard Schölkopf. Measuring statistical dependence with hilbert-schmidt norms. In *International conference on algorithmic learning theory*, pages 63–77. Springer, 2005.

[11] Arthur Gretton, Kenji Fukumizu, Choon Teo, Le Song, Bernhard Schölkopf, and Alex Smola. A kernel statistical test of independence. *Advances in neural information processing systems*, 20, 2007.

[12] Yuhan Hao, Stephanie Hao, Erica Andersen-Nissen, William M Mauck, Shiwei Zheng, Andrew Butler, Maddie J Lee, Aaron J Wilk, Charlotte Darby, Michael Zager, et al. Integrated analysis of multimodal single-cell data. *Cell*, 184(13):3573–3587, 2021.

[13] Jian Hu, Xiangjie Li, Kyle Coleman, Amelia Schroeder, Nan Ma, David J Irwin, Edward B Lee, Russell T Shinohara, and Mingyao Li. Spagcn: Integrating gene expression, spatial location and histology to identify spatial domains and spatially variable genes by graph convolutional network. *Nature methods*, 18(11):1342–1351, 2021.

[14] Xi Jiang, Guanghua Xiao, and Qiwei Li. A bayesian modified ising model for identifying spatially variable genes from spatial transcriptomics data. *Statistics in Medicine*, 41(23):4647–4665, 2022.

[15] Annaliisa Kankainen. *Consistent testing of total independence based on the empirical characteristic function.* PhD thesis, University of Jyvaskyla, 1995.

[16] Sunjong Kwon. Single-molecule fluorescence in situ hybridization: quantitative imaging of single rna molecules. *BMB reports*, 46(2):65, 2013.

[17] Qiwei Li, Minzhe Zhang, Yang Xie, and Guanghua Xiao. Bayesian modeling of spatial molecular profiling data via gaussian process. *Bioinformatics*, 37(22):4129–4136, 2021.

[18] Qi Liu, Chih-Yuan Hsu, and Yu Shyr. Scalable and model-free detection of spatial patterns and colocalization. *Genome research*, 32(9):1736–1745, 2022.

[19] Yaowu Liu, Sixing Chen, Zilin Li, Alanna C Morrison, Eric Boerwinkle, and Xihong Lin. Acat: a fast and powerful p value combination method for rare-variant analysis in sequencing studies. *The American Journal of Human Genetics*, 104(3):410–421, 2019.

[20] Eric Lubeck, Ahmet F Coskun, Timur Zhiyentayev, Mubhij Ahmad, and Long Cai. Single-cell in situ rna profiling by sequential hybridization. *Nature methods*, 11(4):360–361, 2014.

[21] Frank J Massey Jr. The kolmogorov-smirnov test for goodness of fit. *Journal of the American statistical Association*, 46(253):68–78, 1951.

[22] Christopher R Merritt, Giang T Ong, Sarah E Church, Kristi Barker, Patrick Danaher, Gary Geiss, Margaret Hoang, Jaemyeong Jung, Yan Liang, Jill McKay-Fleisch, et al. Multiplex digital spatial profiling of proteins and rna in fixed tissue. *Nature biotechnology*, 38(5):586–599, 2020.

[23] Brendan F Miller, Dhananjay Bambah-Mukku, Catherine Dulac, Xiaowei Zhuang, and Jean Fan. Characterizing spatial gene expression heterogeneity in spatially resolved single-cell transcriptomic data with nonuniform cellular densities. *Genome research*, 31(10):1843–1855, 2021.

[24] Lambda Moses and Lior Pachter. Museum of spatial transcriptomics. *Nature Methods*, 19(5):534–546, 2022.

[25] Carl Edward Rasmussen and Christoopher KI Williams. Gaussian processes for machine learning. *International Journal of Neural Systems*, 14, 2006.

[26] Carl Edward Rasmussen, Christopher KI Williams, et al. *Gaussian processes for machine learning*, volume 1. Springer, 2006.

[27] Samuel G Rodriques, Robert R Stickels, Aleksandrina Goeva, Carly A Martin, Evan Murray, Charles R Vanderburg, Joshua Welch, Linlin M Chen, Fei Chen, and Evan Z Macosko. Slide-seq: A scalable technology for measuring genome-wide expression at high spatial resolution. *Science*, 363(6434):1463–1467, 2019.

[28] Franklin E Satterthwaite. An approximate distribution of estimates of variance components. *Biometrics bulletin*, 2(6):110–114, 1946.

[29] Steven S Skiena. *The algorithm design manual*, volume 2. Springer, 1998.

[30] Robert R Stickels, Evan Murray, Pawan Kumar, Jilong Li, Jamie L Marshall, Daniela J Di Bella, Paola Arlotta, Evan Z Macosko, and Fei Chen. Highly sensitive spatial transcriptomics at near-cellular resolution with slide-seqv2. *Nature biotechnology*, 39(3):313–319, 2021.

[31] Aravind Subramanian, Pablo Tamayo, Vamsi K Mootha, Sayan Mukherjee, Benjamin L Ebert, Michael A Gillette, Amanda Paulovich, Scott L Pomeroy, Todd R Golub, Eric S Lander, et al. Gene set enrichment analysis: a knowledge-based approach for interpreting genome-wide expression profiles. *Proceedings of the National Academy of Sciences*, 102(43):15545–15550, 2005.

[32] Shiquan Sun, Jiaqiang Zhu, and Xiang Zhou. Statistical analysis of spatial expression patterns for spatially resolved transcriptomic studies. *Nature methods*, 17(2):193–200, 2020.

[33] Valentine Svensson, Sarah A Teichmann, and Oliver Stegle. Spatialde: identification of spatially variable genes. *Nature methods*, 15(5):343–346, 2018.

[34] Sanja Vickovic, Gökcen Eraslan, Fredrik Salmén, Johanna Klughammer, Linnea Stenbeck, Denis Schapiro, Tarmo Äijö, Richard Bonneau, Ludvig Bergenstråhle, José Fernandéz Navarro, et al. High-definition spatial transcriptomics for in situ tissue profiling. *Nature methods*, 16(10):987–990, 2019.

[35] Lukas M Weber, Arkajyoti Saha, Abhirup Datta, Kasper D Hansen, and Stephanie C Hicks. nnsvg for the scalable identification of spatially variable genes using nearest-neighbor gaussian processes. *Nature Communications*, 14(1):4059, 2023.

[36] Bernard L Welch. The generalization of 'student's'problem when several different population varlances are involved. *Biometrika*, 34(1-2):28–35, 1947.

[37] Michael C Wu, Arnab Maity, Seunggeun Lee, Elizabeth M Simmons, Quaker E Harmon, Xinyi Lin, Stephanie M Engel, Jeffrey J Molldrem, and Paul M Armistead. Kernel machine snp-set testing under multiple candidate kernels. *Genetic epidemiology*, 37(3):267–275, 2013.

[38] Ke Zhang, Wanwan Feng, and Peng Wang. Identification of spatially variable genes with graph cuts. *Nature Communications*, 13(1):5488, 2022.

[39] Kun Zhang, Jonas Peters, Dominik Janzing, and Bernhard Schölkopf. Kernel-based conditional independence test and application in causal discovery. *arXiv preprint arXiv:1202.3775*, 2012.

[40] Jiaqiang Zhu, Shiquan Sun, and Xiang Zhou. Spark-x: non-parametric modeling enables scalable and robust detection of spatial expression patterns for large spatial transcriptomic studies. *Genome Biology*, 22(1):1–25, 2021.

[41] Qian Zhu, Sheel Shah, Ruben Dries, Long Cai, and Guo-Cheng Yuan. Identification of spatially associated subpopulations by combining scrnaseq and sequential fluorescence in situ hybridization data. *Nature biotechnology*, 36(12):1183–1190, 2018.

[42] Daniel R Zollinger, Stan E Lingle, Kristina Sorg, Joseph M Beechem, and Christopher R Merritt. Geomx™ rna assay: high multiplex, digital, spatial analysis of rna in ffpe tissue. *In Situ Hybridization Protocols*, pages 331–345, 2020.
